# Population-wide cohort study of statin use for the secondary cardiovascular disease prevention in Scotland in 2009–2017

**DOI:** 10.1136/heartjnl-2022-321452

**Published:** 2022-10-03

**Authors:** Inna Thalmann, David Preiss, Iryna Schlackow, Alastair Gray, Borislava Mihaylova

**Affiliations:** 1 Health Economics Research Centre, Nuffield Department of Population Health, University of Oxford, Oxford, UK; 2 MRC Population Health Research Unit, Clinical Trial Service Unit & Epidemiological Studies Unit, Nuffield Department of Population Health, University of Oxford, Oxford, UK; 3 Wolfson Institute of Population Health, Queen Mary University of London, London, UK

**Keywords:** STATINS, Medication Adherence, Quality of Health Care, Coronary Artery Disease, Stroke

## Abstract

**Objective:**

To estimate the extent of suboptimal statin use for the secondary prevention of atherosclerotic cardiovascular disease (ASCVD) at different stages of the treatment pathway and identify patient groups at risk of suboptimal treatment.

**Methods:**

National retrospective cohort study using linked National Health Service Scotland administrative data of adults hospitalised for an ASCVD event (n=167 978) from 2009 to 2017. Proportions of patients initiating, adhering to, discontinuing and reinitiating statins were calculated. We separately examined treatment following myocardial infarction (MI), ischaemic stroke and peripheral arterial disease (PAD) hospitalisations. Multivariable logistic regression and Cox proportional hazards models were used to assess the roles of patient characteristics in the likelihood of initiating and discontinuing statins.

**Results:**

Of patients hospitalised with ASCVD, only 81% initiated statin therapy, 40% of whom used high-intensity statin. Characteristics associated with lower odds of initiation included female sex (28% less likely than men), age below 50 years or above 70 years (<50 year-olds 26% less likely, and 70–79, 80–89 and ≥90 year-olds 22%, 49% and 77% less likely, respectively, than 60–69 year-olds), living in the most deprived areas and history of mental health-related hospital admission. Following MI, 88% of patients initiated therapy compared with 81% following ischaemic stroke and 75% following PAD events. Of statin-treated individuals, 24% discontinued treatment. Characteristics associated with discontinuation were similar to those related to non-initiation.

**Conclusions:**

Statin use remains suboptimal for the secondary ASCVD prevention, particularly in women and older patients, and following ischaemic stroke and PAD hospitalisations. Improving this would offer substantial benefits to population health at low cost.

**WHAT IS ALREADY KNOWN ON THIS TOPIC**

- Despite national clinical guidelines recommending the use of statin for the secondary prevention of atherosclerotic cardiovascular disease (ASCVD), previous studies have found that use of statin therapy among individuals with established ASCVD is suboptimal.
- However, knowledge regarding the extent of, and underlying risk factors for, suboptimal medication use at different treatment stages (namely the initiation of, adherence to, discontinuation of and reinitiation of statin therapy) to inform on the full extent of statin use across the treatment pathway, different populations and disease types, is limited.

**WHAT THIS STUDY ADDS**

- This national study in Scotland, from 2009 to 2017, improves on previous studies by examining in detail all stages of the patient treatment pathway, namely the initiation of, adherence to, discontinuation and reinitiation of statin therapy for the secondary prevention of ASCVD, in order to estimate the full extent of suboptimal statin use across different patient populations and disease types.
- Only 81% of patients initiated statin therapy after hospitalisation for ASCVD and, of those who did, 24% later discontinued statin therapy. Use of intensive statin therapy was also suboptimal.
- Statin treatment following peripheral arterial disease (75% initiated and 29% discontinued statin therapy) and ischaemic stroke (81% and 26%, respectively) was worse than treatment following myocardial infarction (88% and 23%, respectively).
- Women and patients aged <50 years or ≥70 years were systematically undertreated in terms of statin initiation and discontinuation.

**HOW THIS STUDY MIGHT AFFECT RESEARCH, PRACTICE OR POLICY**

- Improvements in use of statin therapy in patients with ASCVD would provide substantial benefits to population health. As many as 6%–10% of subsequent ASCVD events could be avoided.
- Particular focus is needed to improve statin treatment use in women and older patients, and in patients who suffer ischaemic stroke and peripheral arterial disease events, as these individuals remain systematically undertreated.

Individuals with established atherosclerotic cardiovascular disease (ASCVD) are at significantly increased risk of future vascular events.^1^ Statins, a low-density lipoprotein (LDL) cholesterol-lowering therapy, can safely and cost-effectively reduce these risks.^1^ Therefore, national clinical guidelines, such as those issued by the National Institute for Health and Care Excellence (NICE) for England and Wales and the Scottish Intercollegiate Guidelines Network (SIGN) Consortium, have recommended the long-term use of statin therapy for the secondary prevention of ASCVD.

Despite these recommendations, previous studies have found that use of statin therapy among individuals with ASCVD is suboptimal.^2 3^ However, knowledge regarding the extent of, and underlying risk factors for, suboptimal medication use is limited. Policy makers and healthcare providers would benefit from a more detailed understanding of statin use at different treatment stages to inform development of quality improvement programmes.

This study assessed the extent of suboptimal statin therapy use for the secondary prevention of ASCVD in Scotland overall and across subgroups of individuals by age, sex, types of ASCVD and other characteristics. We examined associations between these patient characteristics and statin treatment at different stages of the pathway. This was achieved by using linked and anonymised population-wide administrative National Health Service (NHS) Scotland data for all individuals hospitalised for an ASCVD event in Scotland between October 2009 and July 2017.

## Methods

### Data

NHS Scotland provides comprehensive free healthcare to all people living in Scotland. This retrospective cohort study used large-scale population-wide individual patient data for all individuals hospitalised for ASCVD composed of four linked and anonymised routine healthcare datasets: (1) hospital admissions, (2) specialty mental health admissions, (3) national death records and (4) prescribing information. Individuals were followed between 1 October 2009 and 31 December 2017 (online supplemental table A and figure A).

10.1136/heartjnl-2022-321452.supp1 Supplementary data

### Study population

All Scottish residents aged 18 years or older were included if they had a main discharge diagnosis for an ASCVD event between 1 October 2009 and 3 July 2017, and therefore should have been offered statin treatment according to SIGN’s and NICE’s clinical guidelines for the secondary prevention of cardiovascular disease.^4–7^

The hospital admissions data were used to identify individuals hospitalised for an acute ASCVD event, categorised into myocardial infarction (MI), ischaemic stroke, peripheral arterial disease (PAD) and other ASCVD including events with a hospital length of stay of less than 1 day (online supplemental table B). Following study exclusions (non-Scottish residents, emigration and death within 150 days after discharge, hospital length of stay of >90 days, single medication supply for >365 days and medications in non-pill format; online supplemental figure B), the final study population included 167 978 individuals. Individuals were followed for up to 8 years (average 4.6 years) from the index ASCVD event (recorded on/after 1 October 2009) until study end (ie, 31 December 2017), emigration or death, depending on which event occurred first.

### Primary outcomes: statin initiation, adherence, discontinuation and reinitiation

**Figure 1:**
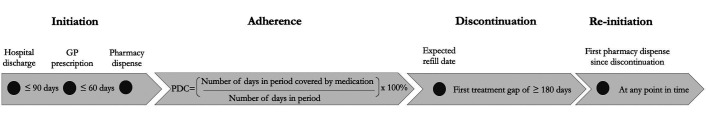
Schematic of the outcome measures initiation, adherence, discontinuation and reinitiation. PDC, proportion of days covered.

Statin therapy use (online supplemental table C) was assessed at four stages of the patient treatment journey: statin initiation, adherence, discontinuation and reinitiation (figure 1). Statin initiation was defined as individuals being prescribed statin therapy within 90 days from index discharge and dispensed within 60 days from that prescription. Adherence was defined as the degree to which patients follow the prescription instructions.^8^ Patients’ prescription records were used to indirectly measure the Proportion of Days Covered (PDC), calculated as the ratio of the number of days the patient is covered by the medication in a period to the total number of days in the period.^9^ Adherence was defined as a PDC threshold of ≥80% and was measured from the date an individual initiated treatment until study end. Discontinuation among patients who initiated statin therapy was measured as the start of the first continuous medication gap of 180 days or more (from the expected medication refill date) since initiation. A binary outcome measure was created to indicate if a patient: (1) discontinued or (2) did not discontinue treatment at any point in time after treatment initiation. The time of the discontinuation event was recorded as the start date of the medication gap. Reinitiation was defined as a record of having been prescribed and dispensed statin therapy at any point in time after the first 180-day treatment gap.

Statins were further grouped into two intensity categories in line with NICE’s definition: statins that reduce low-density lipoprotein cholesterol (LDL-C) by ≤40% and >40% were categorised as low/medium and high intensity, respectively^10^ (online supplemental table D).

### Patient characteristics

The following characteristics were assessed: sex, age at index event date, deprivation quintiles, number of comorbidities, previous mental health hospitalisation, history of previous ASCVD event and/or previous statin use and discharge calendar year (online supplemental table E).

### Statistical analyses

The proportions of individuals who did not initiate therapy, were not adherent, discontinued treatment and did not reinitiate therapy were calculated. Multivariable cross-sectional logistic regression models were used to study the association between patient characteristics and discharge calendar year and the likelihood of initiating statin therapy, and the likelihood of initiating high-intensity statin therapy versus low/medium-intensity therapy. For individuals who initiated statin therapy, a Cox proportional hazards model^11^ of duration from statin initiation (the first day of the first statin therapy dispense after hospital discharge) to discontinuation, censored for death and emigration was used to assess the role of patient characteristics, as well as discharge calendar year, in the discontinuation of statin treatment. A Schoenfeld residuals test for proportional hazards assumption^12^ showed that the proportional hazards assumptions were met.

## Results

Between 2009 and 2017, 167 978 individuals were hospitalised for ASCVD. Baseline demographics at discharge for index ASCVD events are presented in table 1. Demographic characteristics at discharge are presented in online supplemental tables F and G.

**Table 1:** Baseline characteristics of individuals hospitalised for an ASCVD-related event at index discharge

	Total atherosclerotic cardiovascular disease (ASCVD)
N	167 978
Age on discharge, years (mean, SD)	67.4 (12.7)
Female	65 707 (39.1)
Ethnic group	
White	143 578 (85.4)
Other	2373 (1.4)
Missing	22 027 (13.1)
SIMD deprivation quintile (2009)*	
5 (least deprived)	26 294 (15.7)
4	30 896 (18.4)
3	34 330 (20.4)
2	37 290 (22.2)
1 (most deprived)	39 168 (23.3)
ASCVD-related hospitalisation prior to 1 October 2009	67 114 (39.9)
Charlson Comorbidity Index (CCI) (within 12 months prior and incl. index event)	
0 (no comorbidities)†	30 743 (18.3)
1	77 182 (45.9)
2	33 709 (20.1)
3	14 937 (8.9)
4 or more comorbidities	11 407 (6.8)
Mental health inpatient/day case within 12 months prior to index admission	2994 (1.8)
At least one statin prescription in last 12 months prior to index admission‡	93 103 (55.4)
ASCVD type	
MI	50 359 (30)
Stroke	32 873 (20)
PAD	15 149 (9)
Other ASCVD	69 597 (41)

*SIMD is a relative measure of deprivation across Scottish data zones.

†Absence of comorbidities as defined by CCI: in the case of the total ASCVD and other ASCVD populations, this means that individuals were not hospitalised for any of the 17 specified conditions. In the case of the MI, stroke and PAD populations, every individual has at least one CCI comorbidity, their index condition (ie, MI, stroke or PAD); thus, absence of comorbidity is not applicable (N/A).

‡For individuals with index hospitalisations in 2009, information on prior medication use is available from 1 April 2009 and onwards, thereby contributing a minimum of 6 months and up to 12 months of medication history. For all discharges recorded after 1 April 2010, medication history is available for 12 months prior to index admission.

MI, myocardial infarction; PAD, peripheral arterial disease; SIMD, Scottish Index of Multiple Deprivation.

### Statin initiation

**Figure 2:**
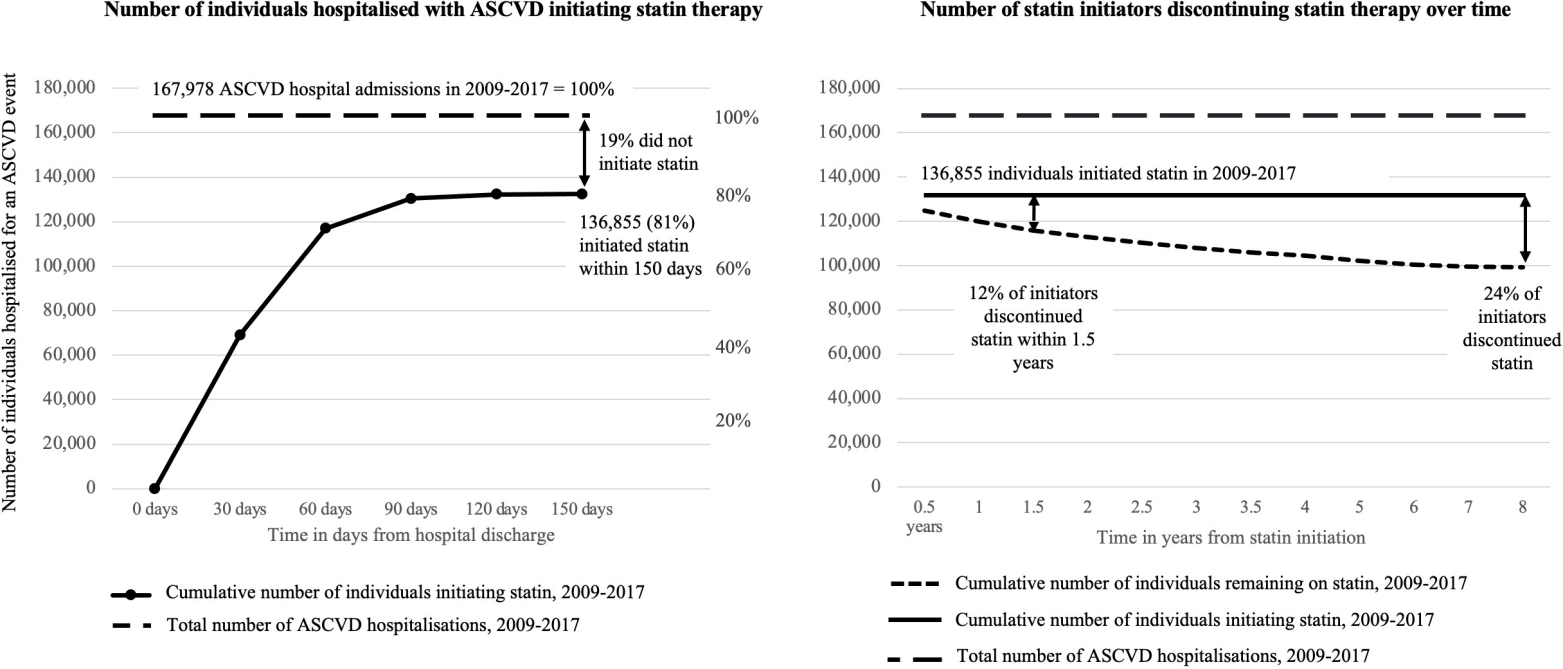
Statin initiation and discontinuation rates following atherosclerotic cardiovascular disease (ASCVD) event.

Out of these individuals, 136 855 (81%) initiated statin therapy (figure 2). Uptake varied significantly by ASCVD type, from 75% among patients following PAD event, to 81% following an ischaemic stroke and 88% of patients following an MI (online supplemental figures C-F). The initiation rate increased little from 80% in 2009–2011 to 82% in 2015–2017.

**Figure 3:**
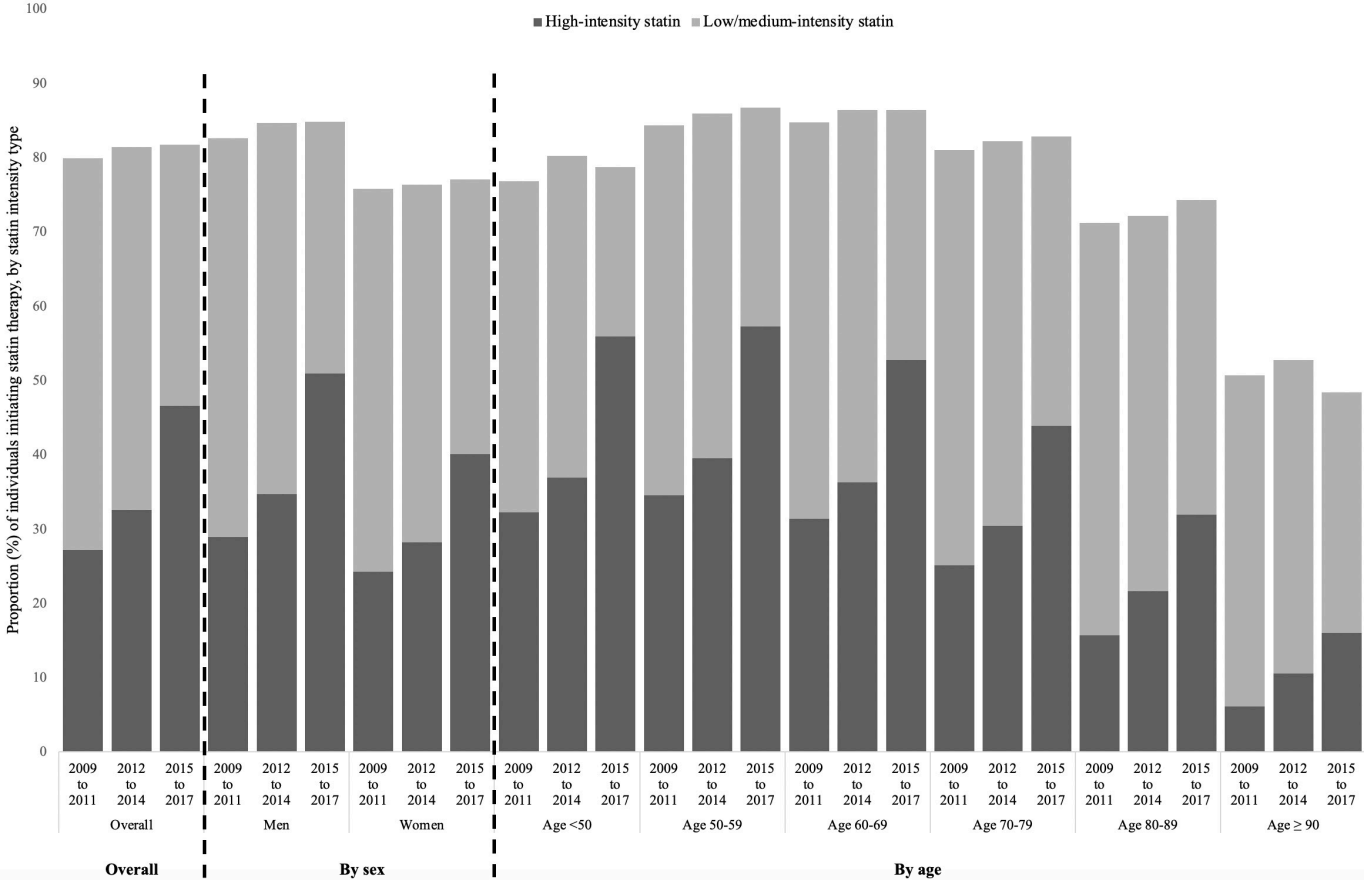
Statin initiation rates following atherosclerotic cardiovascular disease (ASCVD) event in Scotland in 2009–2017, by calendar period, sex, age and statin intensity. There was evidence of an increase in the proportion of individuals initiating any statin over time based on the Cochran-Armitage test for trend (overall: men: p<0.001; women: p<0.5; age <50: p<0.5; age 50-59: p<0.001; age 60-69: p<0.001; age 70-79: p<0.001; age 80-89: p<0.001). There was also strong evidence of an increase in the proportion of statin initiators using high-intensity statin therapy vs. low/medium-intensity therapy (overall and in every sex and age group: p<0.001).

Of patients who initiated statin, 40% initiated high-intensity therapy, with the remainder (60%) prescribed medium or low-intensity statin treatment. Within 12 months from initiation, 5% of initiators uptitrated to high-intensity statin therapy, 2% downtitrated to low/moderate intensity and 93% remained on the intensity initially prescribed. As a result, within 12 months since index prescription, 43% of statin initiators were using high-intensity statin therapy and 57% low/moderate-intensity therapy. The proportion of statin initiators using high-intensity statins within 12 months since index prescription almost doubled since 2009, increasing from 33% in 2009–2011 to 57% in 2015–2017 (p<0.001) (figure 3). High-intensity statin initiation rates in the latest period (ie, 2015–2017) varied significantly by ASCVD type, ranging from 40% for PAD and 51% for stroke to 73% for MI.

**Figure 4:**
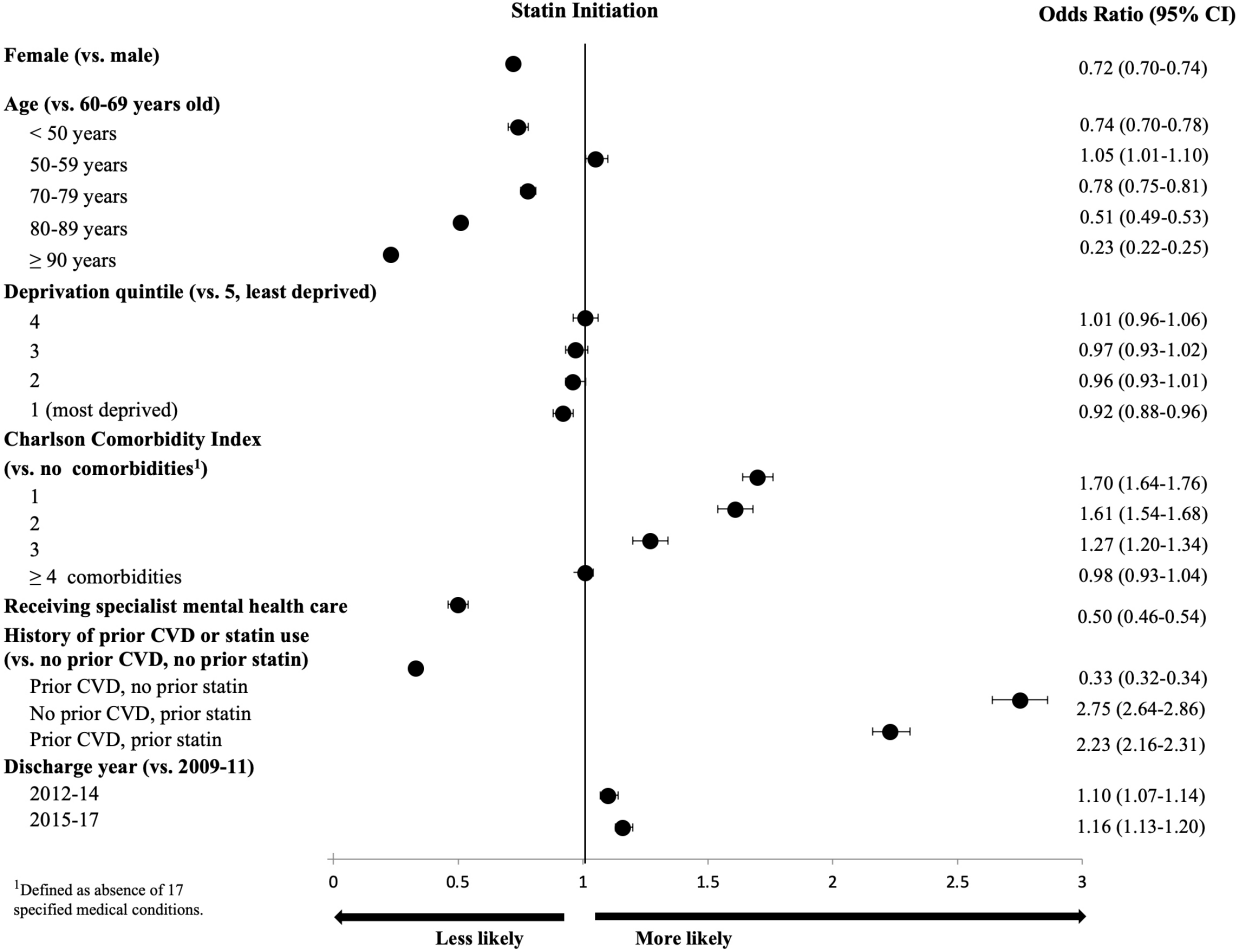
Associations of patient characteristics with statin initiation among individuals with atherosclerotic cardiovascular disease (ASCVD): a multivariable logistic regression model. The Charlson Comorbidity Index (CCI) results presented in this figure are not easily interpretable due to index MI, stroke and PAD included among the CCI-eligible comorbidities, leaving the remaining index ASCVD conditions in the comparator “no comorbidity” category.

Women were 28% less likely to initiate statin than men (OR 0.72 (0.70–0.74)), largely irrespective of age, with 9% lower odds in the case of PAD, 22% for MI and ischaemic stroke and 33% for other atherosclerotic disease. Compared with individuals aged 60–69 years, those below the age of 50 years and those older than 69 years were significantly less likely to initiate statin, with the likelihood of initiation also decreasing as age increased beyond 70 years (<50 years: (OR) 0.74, 95% CI 0.70 to 0.78; 70–79 years: 0.78 (0.75 to 0.81); 80–89 years: 0.51 (0.49 to 0.53); 90 years or older: 0.23 (0.22 to 0.25)). Patients living in the most deprived areas were 8% less likely to initiate statin compared with those in least deprived areas (OR 0.92 (95% CI 0.88 to 0.96)). In addition, those with previous mental health hospitalisations were only half as likely to initiate treatment as those without similar medical histories (OR 0.50 (95% CI 0.46 to 0.54)). A higher number of comorbidities was associated with a lower likelihood of individuals initiating statin. For example, MI and stroke patients with three or more comorbidities had, respectively, 60% and 42% lower odds of initiating statin compared with individuals without comorbidities (MI: OR 0.40 (95% CI 0.37 to 0.44); stroke: OR 0.58 (95% CI 0.53 to 0.64)) (figure 4). Please see online supplemental table H for the associations between CCI and statin initiation by ASCVD type. The same patient characteristics were also associated with lower odds of initiating high-intensity statin therapy compared with low/medium-intensity therapy (online supplemental figure G and table I).

### Statin adherence and discontinuation

While on treatment, 91% of users were adherent. However, 24% of statin users discontinued treatment at some point in time and, of those who discontinued, half (50%) did so within 1.5 years since initiation, and 80% within 3.5 years (figure 2). Discontinuation rates varied by ASCVD type, ranging from 23% for MI patients to 26% for stroke and 29% for PAD patients. Of the individuals who discontinued therapy, only 12 644 individuals (38%) reinitiated therapy at some point in time after their initial treatment gap of 180 days. On average, individuals reinitiated statin about 1.1 years after statin discontinuation.

**Figure 5:**
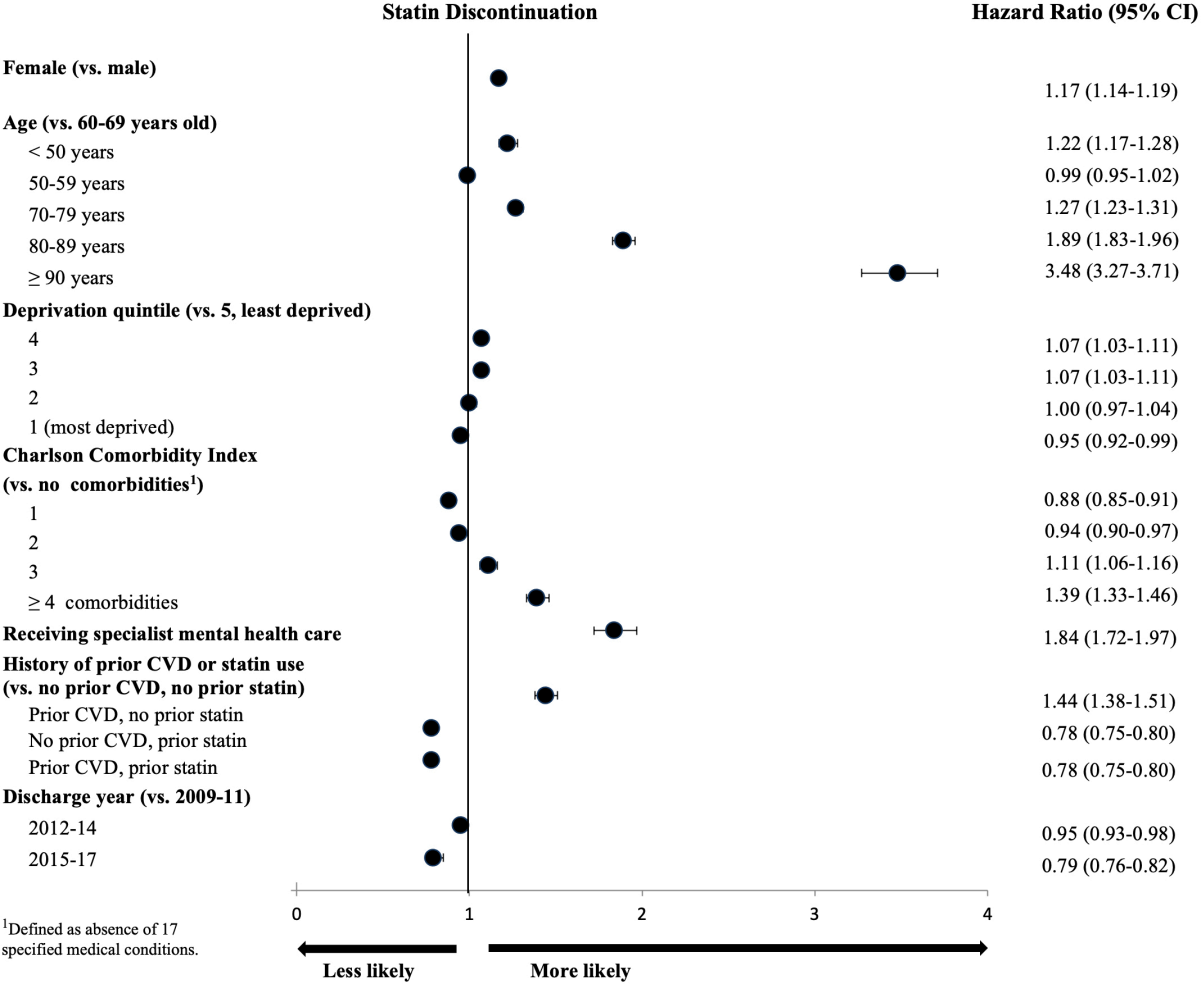
Associations of patient characteristics with statin discontinuation among individuals with atherosclerotic cardiovascular disease (ASCVD): a multivariable Cox proportional hazards model. The Charlson Comorbidity Index (CCI) results presented in this figure are not easily interpretable due to index MI, stroke and PAD included among the CCI-eligible comorbidities, leaving the remaining index ASCVD conditions in the comparator “no comorbidity” category.

Patient characteristics associated with the discontinuation of statin were similar to those related to not initiating therapy. Specifically, women had a 17% higher hazard of statin discontinuation than men (HR 1.17, 95% CI 1.14 to 1.19). A U-shaped association with age was observed: compared with individuals aged 60–69 years, those below the age of 50 years and those aged 70–79 years had, respectively, a 22% (HR 1.22 (95% CI 1.17 to 1.28)) and 27% (HR 1.27 (95% CI 1.23 to 1.31)) higher hazard of discontinuation, with the hazard increasing up to 3.5-fold in the case of patients aged 90 years or older (HR 3.48 (95% CI 3.27 to 3.71)). An increase in the number of comorbidities was associated with an increased hazard of statin discontinuation. For example, MI and stroke patients with three or more concomitant conditions had a 50% higher hazard of statin discontinuation than those without comorbidities (MI: HR 1.50 (95% CI 1.39 to 1.61); stroke: HR 1.47 (95% CI 1.35 to 1.60)) (figure 5; online supplemental table J).

## Discussion

This nationwide study of individuals hospitalised for ASCVD in Scotland demonstrates that use of statin therapy remains suboptimal in these very high-risk individuals. Nineteen per cent of individuals did not initiate statin therapy. While the majority of those who initiated statins were adherent while on treatment, about a quarter (24%) discontinued treatment at some point. Of those patients who stopped treatment, only 38% reinitiated treatment at a later time. Overall, about 25% of patient-years were left untreated after the index ASCVD event. A substantial number of subsequent ASCVD events might therefore have been prevented in these patients with much better statin use. Based on data from the Cholesterol Treatment Trialists’ (CTT) Collaboration (showing that statins yield a proportional reduction in major vascular events of 22% per 1 mmol/L reduction in LDL-C),^1^ about a quarter of subsequent events during untreated patient-years (ie, 6% of all subsequent ASCVD events) might have been prevented if moderate-intensity statins had been used during this time. Up to 40% of major vascular events during untreated patient-years (ie, 10% of all subsequent ASCVD events) might have been prevented if optimal high-intensity statin therapy had been used. This is of at least a similar magnitude to the likely benefit of treating all 167 000 secondary prevention patients with ezetimibe after their index event.^13^ If suboptimal treatment in the same patients extends to other proven secondary prevention interventions, such as antiplatelet agents, beta-blockers and renin–angiotensin system blockers, then the impact of poor treatment use is likely to be far greater.

Our analyses demonstrate that certain groups of patients are at particular risk of suboptimal treatment. Strikingly, women with ASCVD were 28% less likely to commence a statin and 17% more likely to stop than men. Some previous studies have observed that women were less likely than men to be prescribed statin on hospital discharge,^14 15^ but our analyses indicate that suboptimal treatment extends beyond statin initiation. The age of patients was also relevant to the initiation and discontinuation of statin therapy. For example, individuals aged 70–79 years were 22% less likely to initiate statin and had 27% higher hazard of treatment discontinuation compared with those aged 60–69 years. These results were consistent with previous findings of age-related treatment gaps in statin treatment.^16 17^ These important treatment gaps may be due to misperceptions of adverse effects of statins and uncertainty among both patients and prescribers about statin efficacy in women and older patients. However, cardiovascular risk is substantially increased following an ASCVD event, and guidelines are clear in recommending statins to all individuals for the secondary prevention of ASCVD. Given the substantial randomised evidence on statin safety and efficacy for the secondary prevention of CVD in women,^1 18^ and all adult age groups including older patients,^19^ more attention must be paid to the management of these patient groups.

Individuals with a history of hospital admission for specialist mental care, who are known to be at very high cardiovascular risk, had 50% lower odds of initiation and 84% higher hazard of discontinuation of statin therapy compared with individuals without such a history. It is reasonable to propose that patients with milder mental health conditions treated in primary care, such as anxiety and moderate depression, may also be suboptimally treated for the prevention of ASCVD. Mixed findings on the association between mental health and statin use have been reported in previous large studies,^14 20 21^ and further research is required to examine the association between gradients of mental disorders and statin use. Individuals with two or more comorbidities had significantly lower odds of initiating statin and were at higher hazard of discontinuing therapy compared with individuals without physical comorbidities. Previous studies that used large-scale population data on MI patients showed that presence of other diseases was associated with lower odds of statin initiation.^20^

There was also considerable variability in statin initiation and discontinuation rates by ASCVD type. Treatment rates for PAD and ischaemic stroke were poor compared with MI. For instance, only 75% of PAD patients and 81% of stroke patients initiated statin therapy, compared with 88% of MI patients. Among PAD patients 29% discontinued treatment, compared with 26% of stroke and 23% of MI patients. Similar findings were reported in previous observational studies using national registry and prescribing information.^22–24^ Few statin trials were specifically conducted in PAD patients, and current guidelines have been adapted from other at-risk populations such as coronary artery disease, possibly raising uncertainty regarding statin efficacy in PAD.^25^ In the case of secondary prevention in patients who had a stroke, previous studies showed that statins effectively decreased risks of subsequent ischaemic stroke, while potentially increasing risks of haemorrhagic stroke, leading to uncertainty of the overall beneficial effect.^26^ However, evidence from CTT shows that benefits of statin use for secondary prevention heavily outweigh the potential risks of haemorrhagic stroke.^1 27 28^ Therefore, provider training and targeted patient education with a focus on PAD and stroke may narrow the gap with cardiovascular disease types (eg, MI) where guideline recommendations are being followed more rigorously.

A meta-analysis of cardiovascular outcomes trials has shown that the further 0.5 mmol/L reduction in LDL cholesterol obtained with higher intensity statin therapy produces about a 16% further reduction in the incidence of heart attack, revascularisation or ischaemic stroke compared with moderate intensity statin therapy.^1 29^ Use of high-intensity statin therapy improved over time but remained suboptimal, with only 57% of statin initiators in 2015–2017 using high-intensity therapy and with significant variation by ASCVD type (40% of PAD patients, 51% of stroke patients and 73% of MI patients). Alternative and substantially more costly therapies such as PCSK9 inhibitors are considered in patients not reaching LDL-C targets, but findings from this study suggest that there are still considerable opportunities to optimise LDL-C reductions through the use of high-intensity statins.

The dataset has major strengths in that it captures the entire population of Scotland and all healthcare received under a single provider that is free at the point of care and is therefore highly representative. It allowed for a detailed analysis into the extent of statin use. Several limitations should be acknowledged. The data only included prescribing information for individuals who were both prescribed and dispensed treatment in a primary care setting, and therefore, we could not differentiate between treatment not being prescribed or being prescribed but not dispensed. Furthermore, we had data only on medication being collected but not whether patients actually used it, although patients who regularly collect but not use treatment are likely rare. Patient characteristics were derived from hospital records only, which do not capture patients’ full medical history and diagnoses. Similarly, it was not possible to fully account for individuals who were not prescribed or who were asked to discontinue treatment on clinical grounds, such as terminal illness. To mitigate this limitation, the analyses excluded all individuals who died within 150 days of hospital discharge. Lastly, further patient characteristics that may be relevant, such as patients’ ethnicity and marital status, could not be studied due to large numbers of missing observations. The data also cannot provide insights into clinicians’ characteristics and management of a patient, clinicians’ and patients’ beliefs, preferences or risk perceptions of statin therapy.

## Conclusions

The treatment of patients with statin therapy following an ASCVD event remains suboptimal, particularly in women and older patients and following ischaemic stroke and PAD events. Effective approaches to improve statin use are likely to yield important reductions in the burden of cardiovascular disease and at low cost.

## Data Availability

Data may be obtained from a third party and are not publicly available. The data that support the findings of this study are available from Information Services Division (ISD), Public Health Scotland but restrictions apply to the availability of these data, which were used under licence for the current study and so are not publicly available. Data are however available from the authors upon reasonable request and with permission of ISD, Public Health Scotland.
