# Supplementary material for: Population-wide cohort study of statin use for the secondary cardiovascular disease prevention in Scotland in 2009–2017
Source: Heart. 2022 Oct 3;109(5):388–95. doi: 10.1136/heartjnl-2022-321452 (PMC9985707; doi:10.1136/heartjnl-2022-321452)
Supplement: Supplementary data [file heartjnl-2022-321452supp001.pdf]

SUPPLEMENTAL MATERIAL

TABLES

Table A Overview of the four linked NHS Scotland datasets used in this study

Dataset	Examples of information included
General/Acute Inpatient and Day Case (SMR01)	Contains episode-level data on inpatient and day case discharges and covers all individuals (residents and non-residents of Scotland) who receive care in non-obstetric, non-psychiatric NHS general acute specialties and hospitals.<br><br>Each episode contains information on diagnoses using International Classification of Diseases (ICD) codes, operations and procedures, as well as patient and hospital details, such as patient demographics, episode management, socio-economic and geographical information.
Mental Health Inpatient and Day Case (SMR04)	Contains episode-level data on individuals admitted to care in NHS hospital mental health specialties and psychiatric hospitals.<br><br>Each episode contains information on diagnoses using International Classification of Diseases (ICD) codes, as well as patient and hospital details, such as patient demographics, episode management.
National Records of Scotland (NRS deaths)	Contains death-related information (date of death, age and specific causes of death classified under ICD-10)
Prescribing Information System (PIS)	Contains general information on all medicines that were both prescribed and dispensed in the community (i.e., primary care) in Scotland (e.g., information on prescribed items such as formulation code, strength, dose instructions, quantity prescribed; details of the prescribing individuals, practice and dispenser: list size of practice, geographical location). It does not contain information on medicines that were prescribed but not dispensed.

Thalmann I, *et al. Heart* 2023; 109:388–395. doi: 10.1136/heartjnl-2022-321452

Table B Overview of the definitions of ASCVD types by ICD-10 code

ASCVD Type	ICD-10 codes
Total ASCVD	I20-25, I63- 67, I70-71, and I73.9
Myocardial infarction	I21-I22 and min. 1 day hospital length of stay
Ischaemic stroke	I63-I64 and min. 1 day hospital length of stay
Peripheral arterial disease	I71, I73.9
Other ASCVD	I20, I23, I24, I25.0-1, I25.8-9, I65-I67, I70

Table C Overview of Anatomical Therapeutic Chemical (ATC) and British National Formulary (BNF) codes for statin

Statin	ATC code	BNC Code
Atorvastatin	C10AA05	0212000AI
Fluvastatin	C10AA04	0212000M0
Pravastatin	C10AA03	0212000X0
Rosuvastatin	C10AA07	0212000AA
Simvastatin	C10AA01	0212000Y0
Simvastatin & Ezetimibe	-	0212000AC

Table D Definition of high and low/medium statin intensity based on NICE's classification of statin intensity

High-intensity statin	Low-/medium-intensity statin
Atorvastatin 20mg – 80mg	Atorvastatin 10mg
Rosuvastatin 10mg – 40mg	Fluvastatin 20mg – 80mg
Simvastatin 80mg	Pravastatin 10mg – 40mg
	Simvastatin 10mg – 40mg

Statins were grouped into two intensity categories in line with NICE's definition which is based on the percentage reduction in low-density lipoprotein cholesterol (LDL-C) levels: statins that reduce LDL-C by ≤40% and >40% were categorised as low/medium and high intensity, respectively

Thalmann I, *et al. Heart* 2023; 109:388–395. doi: 10.1136/heartjnl-2022-321452

Table E Detailed overview of participant characteristics, and the list of conditions and associated ICD-10 codes included in the Charlson Comorbidity Index (CCI)

The following characteristics were assessed: sex (male, female), age group at index event date (≤49, 50-59, 60-69, 70-79, 80-89, ≥90 years), deprivation quintiles based on the Scottish Index of Multiple Deprivation (where 1 indicates most deprived and 5, least deprived) (11), Charlson Comorbidity Index (defined as the number of comorbidities reported in the hospital admissions data within the 12 months prior to and including index admission discharge, where 'no comorbidities' is defined as the absence of 17 specified medical conditions (see **Supplements, Table E**): no comorbidities, 1, 2, 3, or ≥ 4 comorbidities), inpatient or outpatient admission to a mental health specialty or psychiatric hospital within 12 months prior to index ASCVD admission (yes, no), history of any previous ASCVD event and/or previous statin use in the last 12 months prior to index ASCVD admission (no history of ASCVD and no prior use of statin; history of ASCVD and no prior use of statin; no history of ASCVD and prior use of statin; and history of ASCVD and prior use of statin), and discharge calendar year (2009-2011, 2012-2014, 2015-2017).

**Condition included in the Charlson Comorbidity Index**	**ICD-10 code**
1. AIDS/HIV	B20.X - B22.X, B24.X
2. Any malignancy, including lymphoma and leukaemia, except malignant neoplasm of skin	C00.X - C26.X, C30.X - C34.X, C37.X - C41.X, C43. X, C45. X - C58.X, C60. X - C76.X, C81.X - C85.X, C88.X, C90.X - C97.X
3. Cerebrovascular disease	G45.X, G46.X, H34.0, I60.X - I69.X
4. Chronic pulmonary disease	I27.8, I27.9, J40.X - J47.X, J60.X - J67.X, J68.4, J70.1, J70.3
5. Congestive heart failure	I09.9, I11.0, I13.0, I13.2, I25.5, I42.0, I42.5 - I42.9, I43.X, I50.X, P29.0
6. Dementia	F00.X - F03.X, F05.1, G30.X, G31.1
7. Diabetes without chronic complication	E10.0, E10.1, E10.6, E10.8, E10.9, E11.0, E11.1, E11.6, E11.8, E11.9, E12.0, E12.1, E12.6, E12.8, E12.9, E13.0, E13.1, E13.6, E13.8, E13.9, E14.0, E14.1, E14.6, E14.8, E14.9
8. Diabetes with chronic complication	E10.2 - E10.5, E10.7, E11.2 - E11.5, E11.7, E12.2 - E12.5, E12.7, E13.2 - E13.5, E13.7, E14.2 - E14.5, E14.7
9. Hemiplegia or paraplegia	G04.1, G11.4, G80.1, G80.2, G81.X, G82.X, G83.0 - G83.4, G83.9
10. Metastatic solid tumour	C77.X - C80.X

Thalmann I, *et al. Heart* 2023; 109:388–395. doi: 10.1136/heartjnl-2022-321452

**Condition included in the Charlson Comorbidity Index**	**ICD-10 code**
11. Mild liver disease	B18.X, K70.0 - K70.3, K70.9, K71.3 - K71.5, K71.7, K73.X, K74.X, K76.0, K76.2 - K76.4, K76.8, K76.9, Z94.4
12. Moderate or severe liver disease	I85.0, I85.9, I86.4, I98.2, K70.4, K71.1, K72.1, K72.9, K76.5, K76.6, K76.7
13. Myocardial infarction	I21.X, I22.X, I25.2
14. Peptic ulcer disease	K25.X - K28.X
15. Peripheral vascular disease	I70.X x, I71.X, I73.1, I73.8, I73.9, I77.1, I79.0, I79.2, K55.1, K55.8, K55.9, Z95.8, Z95.9
16. Renal disease	I12.0, I13.1, N03.2 - N03.7, N05.2 - N05.7, N18.X, N19.X, N25.0, Z49.0 - Z49.2, Z94.0, Z99.2
17. Rheumatic disease	M05.X, M06.X, M31.5, M32.X - M34.X, M35.1, M35.3, M36.0

Thalmann I, *et al. Heart* 2023; 109:388–395. doi: 10.1136/heartjnl-2022-321452

Table F Participant characteristics of statin initiators and non-initiators at index admission discharge, total ASCVD and by ASCVD type

	**Total Atherosclerotic cardiovascular disease**		**Myocardial infarction**		**Ischaemic stroke**		**Peripheral arterial disease**		**Other ASCVD**	
Statin initiation	Yes	No	Yes	No	Yes	No	Yes	No	Yes	No
**N (%)**	136,855 (81.5)	31,123 (18.5)	44,440 (88.3)	5,919 (11.8)	26,501 (80.6)	6,372 (19.4)	11,364 (75.0)	3,785 (25.0)	54,550 (78.4)	15,047 (21.6)
**Age on discharge, years** (mean, SD)	66.8 (12.2)	70.0 (14.9)	64.9 (13.1)	73.6 (14.5)	70.1 (12.8)	73.7 (15.4)	68.9 (10.5)	67.9 (14.9)	66.3 (10.9)	67.6 (14.2)
**Hospital length of stay, days** (mean, SD)	6.8 (12.1)	9.5 (16.1)	6.1 (6.9)	9.8 (12.1)	15.2 (19.6)	21.8 (23.2)	8.3 (12.9)	10.2 (14.6)	2.9 (7.4)	4.0 (10.2)
**Female**	36.8	49.1	33.6	47.9	46.3	56.3	33.4	40.6	35.6	48.7
**Ethnic group**										
White	85.3	86.3	85.5	87.6	85.2	86.0	86.6	86.6	84.8	86.0
Other	1.4	1.3	1.7	1.5	0.9	0.8	0.3	0.6	1.7	1.5
Missing	13.3	12.4	12.8	10.9	13.9	13.2	13.1	12.8	13.5	12.5
**SIMD quintile (2009)**[1]										
5 (least deprived)	15.7	15.5	15.3	15.2	15.8	16.2	13.3	13.8	16.4	15.8
4	18.5	18.1	18.5	17.0	17.8	17.6	18.4	19.1	18.7	18.6
3	20.4	20.6	20.3	21.2	19.8	19.5	21.4	20.9	20.5	20.7
2	22.2	22.3	22.1	22.8	22.2	22.9	24.2	23.1	21.8	21.6
1 (most deprived)	23.3	23.5	23.7	23.8	24.4	23.8	22.8	23.1	22.5	23.3
**Urban-Rural Classification**[2]										
Large urban areas	31.9	33.3	32.3	35.8	33.7	34.4	27.9	27.1	31.8	33.4
Other urban areas	36.1	35.8	36.0	34.3	35.7	35.5	37.7	40.2	36.1	35.4
Accessible small towns	9.7	9.0	9.8	9.1	9.7	8.8	9.5	9.6	9.6	8.9
Remote small towns	3.9	4.2	3.9	4.5	3.7	4.6	4.8	3.7	4.0	4.1
Accessible rural	11.6	11.2	11.7	10.5	11.2	10.5	12.3	12.9	11.5	11.5
Remote rural	6.7	6.4	6.4	5.8	5.6	6.3	7.7	6.6	7.1	6.7
**Charlson Comorbidity Index**										

[1] Scottish Index of Multiple Deprivation (SIMD) is a relative measure of deprivation across Scottish data zones.
[2] Scottish Government Urban Rural Classification: (1) Large urban areas: settlements of 125,0000 or more people; (2) Other urban areas: settlements of 10,000 to 124,999 people; (3) Accessible small towns: settlements of 3,000 to 9,999 people and within 30 minutes' drive of a settlement of 10,000 or more; (4) Remote small towns: settlements of 3,000 to 9,999 people and a drive time of over 30 minutes to a settlement of 10,000 or more; (5) Accessible rural: Areas with a population of less than 3,000 people, and within a 30 minute drive time from a settlement of 10,000 or more; (6) Remote rural: Areas with a population of less than 3,000 people, and with a drive time of over 30 minutes to a settlement of 10,000 or more.

Thalmann I, *et al. Heart* 2023; 109:388–395. doi: 10.1136/heartjnl-2022-321452

	**Total Atherosclerotic cardiovascular disease**		**Myocardial infarction**		**Ischaemic stroke**		**Peripheral arterial disease**		**Other ASCVD**	
Statin initiation	Yes	No	Yes	No	Yes	No	Yes	No	Yes	No
0 (no-comorbidities)[3]	18.0	19.3	N/A	N/A	N/A	N/A	N/A	N/A	45.1	40.9
1	47.1	41.4	54.9	36.8	57.8	50.6	62.9	62.2	32.2	33.2
2	20.3	19.2	27.6	29.6	23.0	24.1	17.9	17.8	13.4	13.4
3	8.5	10.5	10.2	18.2	11.3	13.7	10.5	10.0	5.4	6.9
4 or more comorbidities	6.1	9.6	7.3	17.4	7.9	11.7	8.7	9.9	3.8	5.5
**Mental health inpatient/day case 12 months prior to index admission**	1.5	3.1	1.3	3.5	2.1	4.4	1.1	1.9	1.4	2.9
**ASCVD history 12 months prior to index admission**[4]										
No ASCVD hospitalisation + no statin use	33.9	41.9	54.1	40.5	45.5	47.8	10.8	48.5	16.6	38.3
ASCVD hospitalisation + no statin use	5.6	25.2	7.1	25.4	8.1	20.5	3.6	29.6	3.5	26.0
No ASCVD hospitalisation + statin use	27.5	12.3	17.1	10.8	23.2	13.3	36.4	9.1	36.1	13.3
ASCVD hospitalisation + statin use	33.1	20.6	21.7	23.3	23.2	18.4	49.2	12.8	43.7	22.4
**Time to first primary care level prescription since index discharge, days** (mean, SD)[5]	21.4 (19.3)	Not applicable	17.5 (16.8)	Not applicable	18.8 (18.3)	Not applicable	27.6 (21.6)	Not applicable	24.7 (20.2)	Not applicable
**Time from prescription to dispense, days** (mean, SD)	11.1(13.4)		10.7 (16.9)		12.5 (14.9)		11.1 (13.1)		10.8 (12.9)	
**Statin intensity**[6]										
High intensity	40.4		49.4		33.9		31.3		37.3	
Moderate/low intensity	59.6		50.6		66.1		68.7		62.7	

[3] Absence of comorbidities as defined by CCI: in the case of the total ASCVD and other ASCVD populations, this means that individuals were not hospitalised for any of the 17 specified conditions. In the case of the MI, stroke and PAD populations, every individual has at least one CCI comorbidity, their index condition (i.e. MI, stroke or PAD), thus absence of comorbidity is not applicable (N/A).

[4] For individuals with index hospitalisations in 2009, information on prior medication use is available from 1 April 2009 and onward, thereby contributing a minimum of 6 months and up to 12 months of medication history. For all discharges recorded after 1 April 2010, medication history is available for 12 months prior to index admission.

[5] Please note that this statistic only includes individuals who initiated the prescribed treatment, as the Prescribing Information System (PIS) data do not contain prescribing information for patients who were not dispensed treatment.

[6] In line with NICE's categorisation of statin intensity, high-intensity statins are defined as statins that reduce LDL-C levels by >40%, while moderate/low-intensity statins reduce LDL-C levels by ≤40%.

Thalmann I, *et al. Heart* 2023; 109:388–395. doi: 10.1136/heartjnl-2022-321452

Table G Characteristics of patients who did and did not discontinue statin therapy, total ASCVD and by ASCVD type

	**Total Atherosclerotic cardiovascular disease**		**Myocardial infarction**		**Ischaemic stroke**		**Peripheral arterial disease**		**Other ASCVD**	
Statin discontinuation	Yes	No	Yes	No	Yes	No	Yes	No	Yes	No
**N (%)**	32,983 (24.1)	103,872 (75.9)	10,069 (22.7)	34,371 (77.3)	6,802 (25.7)	19,699 (74.3)	3,343 (29.4)	8,021 (70.6)	12,769 (23.4)	41,781 (76.6)
**Age on discharge** (mean, SD)	68.5 (13.2)	66.3 (11.8)	67.4 (14.3)	64.2 (12.6)	71.6 (13.8)	69.5 (12.3)	69.8 (11.3)	68.7 (10.2)	67.3 (12.0)	66.1 (10.6)
**Deaths during follow-up time**	35.4	14.3	32.8	13.1	44.2	10.1	48.7	22.3	29.2	11.6
**Age at death** (mean, SD)	79.1 (10.4)	77.8 (10.1)	79.5 (10.9)	78.1 (10.6)	81.4 (9.9)	79.9 (9.8)	77.0 (9.6)	76.1 (9.3)	77.7 (10.3)	76.6 (9.8)
**Time to death since index discharge** (mean, SD)	3.2 years (1.9)	2.8 years (1.8)	3.1 years (1.9)	2.6 years (1.8)	3 years (1.8)	2.6 years (1.7)	3.1 years (1.8)	4.6 years (1.9)	3.4 years (1.9)	3 years (1.9)
**Time to discontinuation** (mean, SD)	2 years (1.7)	Not applicable	2 years (1.7)	Not applicable	1.9 years (1.7)	Not applicable	2 years (1.7)	Not applicable	2.1 years (1.8)	Not applicable
**Female**	41.6	35.4	39.7	31.8	50.0	45.0	35.7	32.4	40.2	34.2
**Ethnic group**										
White	86.2	85.0	86.4	85.3	86.3	84.9	86.4	86.7	86.1	84.4
Other	1.5	1.4	1.9	1.6	0.9	0.9	0.3	0.4	1.8	1.7
Missing	12.3	13.6	11.7	13.1	12.8	14.2	13.3	12.9	12.2	13.8
**SIMD quintile (2009)**[7]										
5 (least deprived)	15.4	15.8	14.7	15.5	16.5	15.6	12.6	13.5	16.0	16.6
4	19.4	18.1	19.3	18.3	19.7	17.2	19.1	18.1	19.4	18.5
3	21.3	20.1	21.3	20.0	20.9	19.4	23.0	20.8	21.1	20.4
2	22.1	22.2	21.9	22.2	21.5	22.5	23.0	24.6	22.3	21.7
1 (most deprived)	21.8	23.8	22.7	24.0	21.5	25.4	22.2	23.0	21.2	22.9
**Urban-Rural Classification**[8]										
Large urban areas	31.6	32.2	32.1	32.3	33.1	33.9	26.9	28.4	30.5	32.2
Other urban areas	34.4	36.7	33.8	36.6	33.0	36.7	37.5	37.8	34.9	36.4
Accessible small towns	9.7	9.7	9.7	9.9	10.0	9.6	9.0	9.7	9.8	9.5
Remote small towns	4.7	3.8	4.7	3.7	4.5	3.5	5.6	4.5	4.6	3.8

[7] Scottish Index of Multiple Deprivation (SIMD) is a relative measure of deprivation across Scottish data zones.
[8] Scottish Government Urban Rural Classification: (1) Large urban areas: settlements of 125,0000 or more people; (2) Other urban areas: settlements of 10,000 to 124,999 people; (3) Accessible small towns: settlements of 3,000 to 9,999 people and within 30 minutes' drive of a settlement of 10,000 or more; (4) Remote small towns: settlements of 3,000 to 9,999 people and a drive time of over 30 minutes to a settlement of 10,000 or more; (5) Accessible rural: Areas with a population of less than 3,000 people, and within a 30 minute drive time from a settlement of 10,000 or more; (6) Remote rural: Areas with a population of less than 3,000 people, and with a drive time of over 30 minutes to a settlement of 10,000 or more.

	**Total Atherosclerotic cardiovascular disease**		**Myocardial infarction**		**Ischaemic stroke**		**Peripheral arterial disease**		**Other ASCVD**	
Statin discontinuation	Yes	No	Yes	No	Yes	No	Yes	No	Yes	No
Accessible rural	12.1	11.4	11.9	11.6	12.1	10.9	12.6	12.2	12.1	11.3
Remote rural	7.9	6.3	7.7	5.9	7.3	5.5	8.5	7.4	8.1	6.8
**Charlson Comorbidity Index**										
0 (no-comorbidities) [9]	17.7	18.0	N/A	N/A	N/A	N/A	N/A	N/A	45.8	44.9
1	44.6	47.9	49.3	56.5	56.2	58.4	59.9	64.1	30.6	32.7
2	20.0	20.4	28.6	27.4	22.6	22.1	17.1	18.2	12.6	13.7
3	9.8	8.1	12.3	9.6	11.8	11.1	12.4	9.7	6.1	5.2
4 or more comorbidities	7.9	5.6	9.8	6.5	9.5	7.4	10.5	8.0	4.8	3.5
**Mental health inpatient/day case 12 months prior to index admission**	2.8	1.1	2.4	0.9	3.6	1.5	2.0	0.8	2.8	1.0
**ASCVD history in the 12 months prior to index admission[10]**										
No CVD hospitalisation + no statin	34.6	33.7	51.1	54.9	46.7	45.0	13.0	9.9	20.8	15.4
No CVD hospitalisation + statin use	23.4	28.8	14.1	18.0	19.0	24.8	32.8	37.8	30.6	37.8
CVD hospitalisation + no statin use	8.4	4.6	10.8	6.1	10.6	7.2	6.1	2.6	6.1	2.7
CVD hospitalisation + statin use	33.6	32.9	4.0	21.0	23.8	22.9	48.1	49.7	42.5	44.2

[9] Absence of comorbidities as defined by CCI: in the case of the total ASCVD and other ASCVD populations, this means that individuals were not hospitalised for any of the 17 specified conditions. In the case of the MI, stroke and PAD populations, every individual has at least one CCI comorbidity, their index condition (i.e. MI, stroke or PAD), thus absence of comorbidity is not applicable (N/A).

[10] For individuals with index hospitalisations in 2009, information on prior medication use is available from 1 April 2009 and onward, thereby contributing a minimum of 6 months and up to 12 months of medication history. For all discharges recorded after 1 April 2010, medication history is available for 12 months prior to index admission.

Thalmann I, *et al. Heart* 2023; 109:388–395. doi: 10.1136/heartjnl-2022-321452

Table H Associations of patient characteristics with statin therapy initiation among individuals with ASCVD, by CVD type (multivariable logistic regression models)

	Myocardial infarction		Ischaemic stroke		Peripheral arterial disease		Other ASCVD	
	OR	95% CI	OR	95% CI	OR	95% CI	OR	95% CI
**Female (vs. male)**	0.77***	0.73-0.82	0.79***	0.75-0.84	0.91	0.84-1.00	0.67***	0.64-0.70
**Age (vs. 60-69 years old)**								
<50	0.98	0.87-1.10	0.55***	0.49-0.62	0.60***	0.51-0.71	0.63***	0.59-0.69
50-59 years	1.18**	1.06-1.32	0.99	0.88-1.12	0.89	0.78-1.02	0.96	0.90-1.02
70-79 years	0.66***	0.60-0.72	0.71***	0.65-0.78	1.00	0.90-1.11	0.82***	0.77-0.87
80-80 years	0.34***	0.31-0.37	0.47***	0.43-0.52	0.78***	0.68-0.88	0.51***	0.48-0.54
≥ 90 years	0.13***	0.11-0.15	0.20***	0.17-0.23	0.50***	0.37-0.66	0.23***	0.20-0.27
**Deprivation quintile (vs. 5, least deprived)**								
4	1.08	0.97-1.19	1.05	0.95-1.16	1.02	0.88-1.18	0.99	0.92-1.06
3	0.94	0.85-1.04	1.03	0.93-1.13	1.08	0.95-1.25	0.98	0.91-1.05
2	0.95	0.86-1.04	0.97	0.88-1.07	1.07	0.94-1.23	1.00	0.93-1.07
1	0.88*	0.80-0.97	0.94	0.86-1.03	0.94	0.82-1.08	0.94	0.88-1.00
**Charlson Comorbidity Index (vs. no comorbidities)[1]**								
1	N/a	N/a	N/a	N/a	N/a	N/a	0.99	0.94-1.04
2	0.71***	0.66-0.76	0.78***	0.73-0.84	0.93	0.83-1.04	0.96	0.90-1.03
3	0.56***	0.51-0.61	0.72***	0.66-0.79	0.91	0.79-1.05	0.70***	0.64-0.77
4 or more	0.40***	0.37-0.44	0.58***	0.53-0.64	0.67***	0.58-0.78	0.63***	0.57-0.70
**Receiving specialist mental health care**	0.40***	0.33-0.48	0.49***	0.40-0.54	0.75	0.54-1.05	0.53***	0.46-0.61
**History of prior CVD or statin use (vs. no prior CVD, no prior statin)**								
Prior CVD, no prior statin	0.34***	0.31-0.37	0.52***	0.48-0.57	0.55***	0.48-0.63	0.35***	0.32-0.37
No prior CVD, prior statin	1.53***	1.39-1.68	1.97***	1.81-2.14	7.98***	7.14-8.92	5.93***	5.59-6.28
Prior CVD, prior statin	1.24***	1.15-1.35	1.63***	1.51-1.77	7.84***	7.06-8.72	5.00***	4.74-5.27
**Discharge year (vs. 2009-11)**								
2012-14	1.10**	1.03-1.18	1.08*	1.00-1.15	1.08	0.98-1.19	1.06*	1.01-1.11
2015-17	1.11**	1.03-1.19	1.29***	1.20-1.39	0.96	0.86-1.06	1.14***	1.08-1.20

Note: (***): p<0.001; (**): p<0.01; (*): p<0.5; [1] In the case of myocardial infarction, stroke and peripheral arterial disease, the reference group is 'one comorbidity' as the Charlson Comorbidity Index classifies the presence of these conditions as a comorbidity.

Thalmann I, *et al. Heart* 2023; 109:388–395. doi: 10.1136/heartjnl-2022-321452

Table I Associations of patient characteristics with high-intensity statin therapy initiation (vs. moderate/low intensity therapy) among individuals with ASCVD, by CVD type (multivariable logistic regression models)

	**Myocardial infarction**		**Ischaemic stroke**		**Peripheral arterial disease**		**Other ASCVD**	
	OR	95% CI	OR	95% CI	OR	95% CI	OR	95% CI
**Female (vs. male)**	0.87***	0.83-0.91	1.00	0.94-1.05	1.16**	1.06-1.26	1.00	0.97-1.04
**Age (vs. 60-69 years old)**								
<50	1.32***	1.23-1.42	0.98	0.87-1.10	0.88	0.71-1.09	1.28***	1.19-1.38
50-59 years	1.17***	1.10-1.24	1.07	0.98-1.17	1.09	0.96-1.24	1.16***	1.11-1.22
70-79 years	0.73***	0.69-0.78	0.79***	0.73-0.85	0.86**	0.78-0.95	0.76***	0.73-0.80
80-80 years	0.43***	0.40-0.46	0.57***	0.53-0.62	0.54***	0.47-0.61	0.52***	0.49-0.56
≥ 90 years	0.24***	0.20-0.28	0.35***	0.29-0.42	0.42***	0.27-0.66	0.28***	0.22-0.36
**Deprivation quintile (vs. 5, least deprived)**								
4	1.09*	1.02-1.17	1.11*	1.01-1.22	1.01	0.87-1.17	0.95	0.90-1.01
3	1.13***	1.05-1.21	1.04	0.95-1.14	0.99	0.85-1.14	0.99	0.93-1.05
2	1.00	0.93-1.07	1.04	0.95-1.14	1.09	0.95-1.26	1.02	0.96-1.08
1	0.93*	0.87-0.99	1.00	0.92-1.09	1.03	0.90-1.19	0.98	0.93-1.04
**Charlson Comorbidity Index (vs. no comorbidities)[1]**								
1	N/A	N/A	N/A	N/A	N/A	N/A	1.33***	1.27-1.39
2	0.88***	0.84-0.92	1.15***	1.07-1.23	1.14*	1.02-1.27	1.50***	1.42-1.58
3	0.77***	0.71-0.82	1.09	1.00-1.19	1.18*	1.03-1.35	1.55***	1.43-1.68
4 or more	0.79***	0.73-0.86	1.14*	1.03-1.26	1.13	0.98-1.31	1.53***	1.39-1.68
**Receiving specialist mental health care**	0.76**	0.63-0.92	0.72**	0.58-0.88	0.72	0.48-1.09	0.92	0.79-1.07
**History of prior CVD or statin use (vs. no prior CVD, no prior statin)**								
Prior CVD, no prior statin	0.89**	0.82-0.97	1.08	0.96-1.20	1.11	0.84-1.29	1.02	0.91-1.14
No prior CVD, prior statin	1.44***	1.36-1.52	2.16***	2.02-2.32	1.60***	1.36-1.87	1.15***	1.09-1.22
Prior CVD, prior statin	1.83***	1.73-1.94	2.71***	2.52-2.92	2.76***	2.36-3.23	2.02***	1.91-2.13
**Discharge year (vs. 2009-11)**								
2012-14	1.44***	1.38-1.51	1.54***	1.44-1.66	1.25***	1.13-1.37	1.17***	1.12-1.22
2015-17	6.35***	6.01-6.71	4.11***	3.83-4.41	1.86***	1.67-2.07	2.38**	2.27-2.49

Note: (***): p<0.001; (**): p<0.01; (*): p<0.5; [1] In the case of myocardial infarction, stroke and peripheral arterial disease, the reference group is 'one comorbidity' as the Charlson Comorbidity Index classifies the presence of these conditions as a comorbidity.

Thalmann I, *et al. Heart* 2023; 109:388–395. doi: 10.1136/heartjnl-2022-321452

Table J Associations of patient characteristics with statin therapy discontinuation among individuals with ASCVD, by CVD type (multivariable Cox proportional hazards models)

	**Myocardial infarction**		**Ischaemic stroke**		**Peripheral arterial disease**		**Other ASCVD**	
	HR	95% CI	HR	95% CI	HR	95% CI	HR	95% CI
**Female (vs. male)**	1.21***	1.16-1.26	1.09***	1.04-1.15	1.09*	1.02-1.17	1.19***	1.15-1.23
**Age (vs. 60-69 years old)**								
<50	1.18***	1.10-1.27	1.16***	1.45-1.78	1.26**	1.06-1.49	1.35***	1.26-1.45
50-59 years	0.96	0.90-1.03	1.11*	1.01-1.21	1.10	0.98-1.23	1.03	0.97-1.08
70-79 years	1.31***	1.23-1.38	1.29***	1.20-1.39	1.18***	1.08-1.29	1.23***	1.17-1.29
80-80 years	2.02***	1.89-2.15	1.92***	1.78-2.07	1.78***	1.60-1.97	1.85***	1.72-1.93
≥ 90 years	3.93***	3.53-4.37	3.45***	3.08-3.88	2.87***	2.27-3.63	3.86***	3.39-4.39
**Deprivation quintile (vs. 5, least deprived)**								
4	1.09**	1.02-1.16	1.05	0.97-1.13	1.10	0.97-1.24	1.07*	1.01-1.13
3	1.10**	1.03-1.18	0.99	0.92-1.07	1.14*	1.01-1.28	1.06*	1.00-1.12
2	1.02	0.95-1.09	0.89**	0.82-0.96	1.00	0.88-1.12	1.05	0.99-1.11
1	1.01	0.94-1.08	0.83***	0.77-0.90	1.06	0.94-1.20	0.97	0.91-1.03
**Charlson Comorbidity Index (vs. no comorbidities)[1]**								
1	N/a	N/a	N/a	N/a	N/a	N/a	0.94**	0.90-0.97
2	1.08**	1.03-1.13	1.09**	1.03-1.16	1.02	0.93-1.12	0.95	0.90-1.00
3	1.24***	1.16-1.32	1.14**	1.05-1.23	1.38***	1.24-1.53	1.15***	1.07-1.24
4 or more	1.50***	1.39-1.61	1.47***	1.35-1.60	1.60***	1.42-1.79	1.46***	1.34-1.58
**Receiving specialist mental health care**	1.81***	1.59-2.05	1.80***	1.59-2.05	1.69***	1.34-2.13	1.95***	1.76-2.16
**History of prior CVD or statin use (vs. no prior CVD, no prior statin)**								
Prior CVD, no prior statin	1.36***	1.27-1.45	1.20***	1.11-1.30	1.14*	1.01-1.30	0.98	0.92-1.06
No prior CVD, prior statin	1.01	0.95-1.08	0.96	0.90-1.03	0.94	0.84-1.04	0.79***	0.75-0.83
Prior CVD, prior statin	1.05	0.99-1.10	0.98	0.92-1.05	0.84**	0.76-0.92	0.70***	0.67-0.74
**Discharge year (vs. 2009-11)**								
2012-14	0.97	0.93-1.01	0.91**	0.87-0.96	0.96	0.89-1.04	0.98	0.95-1.02
2015-17	0.83***	0.78-0.88	0.78***	0.73-0.84	0.83**	0.74-0.93	0.77***	0.72-0.81

Note: (***): p<0.001; (**): p<0.01; (*): p<0.5; [1] In the case of myocardial infarction, stroke and peripheral arterial disease the reference group is 'one comorbidity' as the Charlson Comorbidity Index classifies the presence of these conditions as a comorbidity.

Thalmann I, *et al. Heart* 2023; 109:388–395. doi: 10.1136/heartjnl-2022-321452

FIGURES

Figure A Overview of the linkage and study population

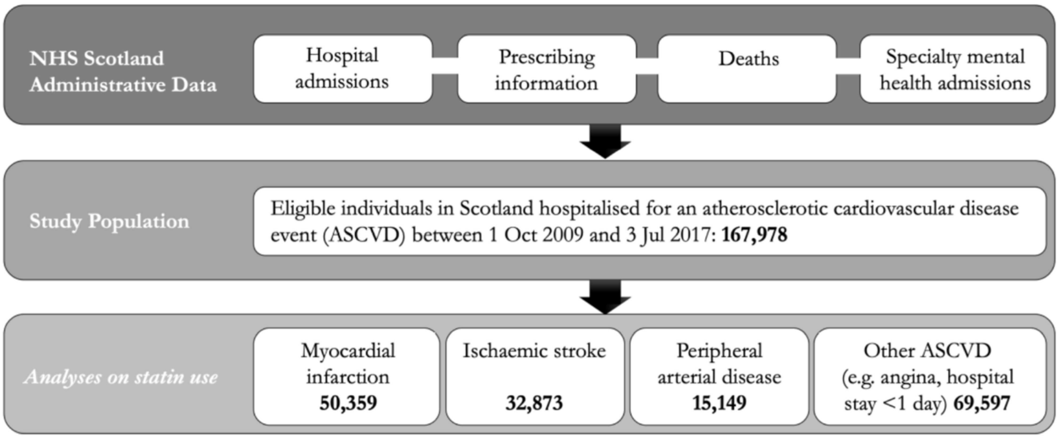

Figure B Flowchart of the study population

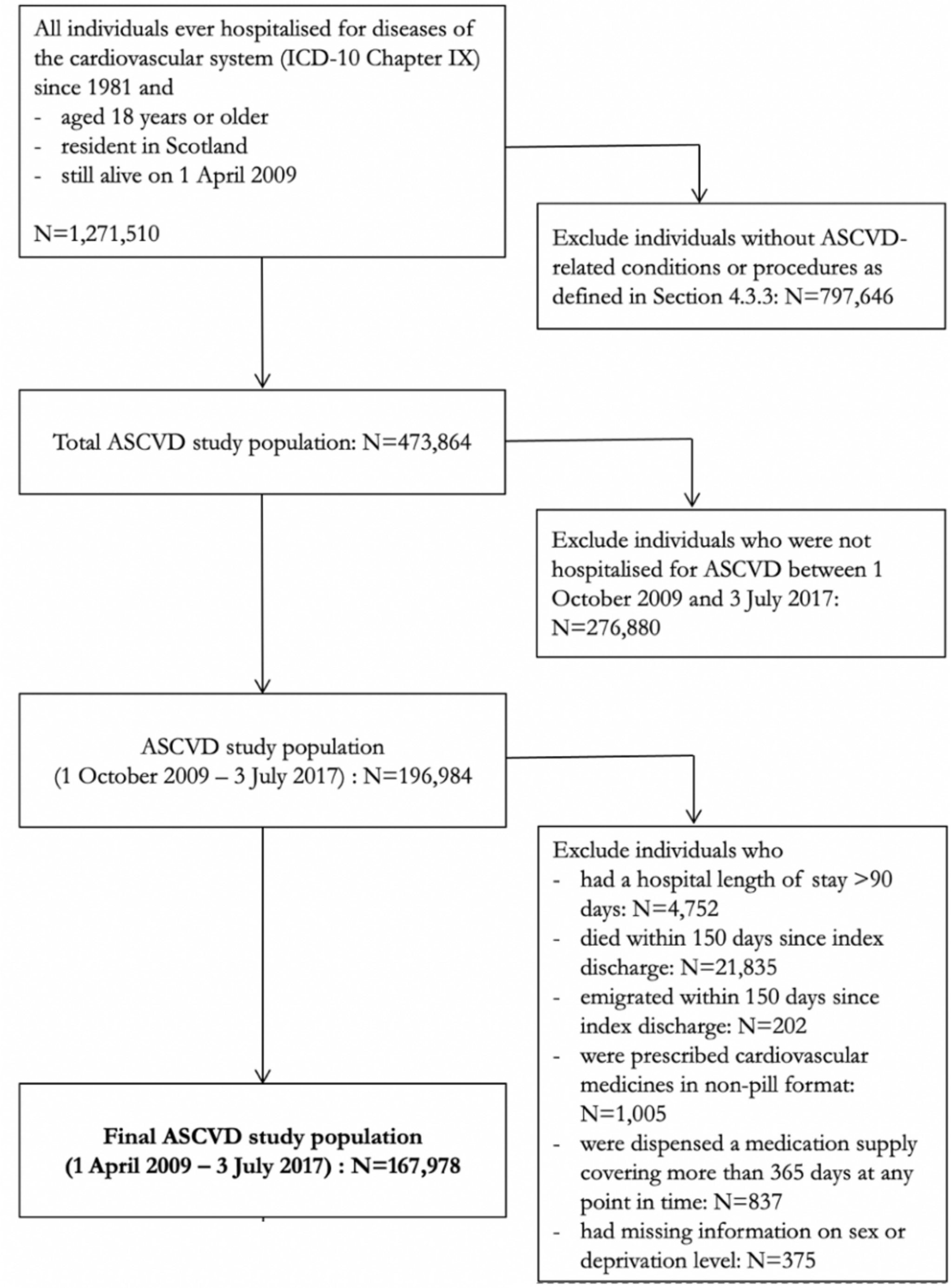

Thalmann I, *et al. Heart* 2023; 109:388–395. doi: 10.1136/heartjnl-2022-321452

Figure C Statin initiation and discontinuation rates over time following myocardial infarction event

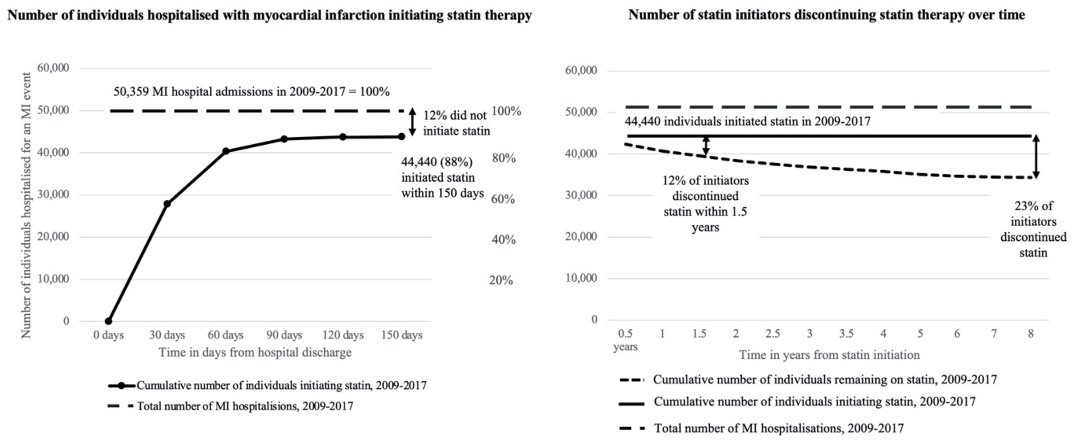

Figure D Statin initiation and discontinuation rates over time following ischaemic stroke event

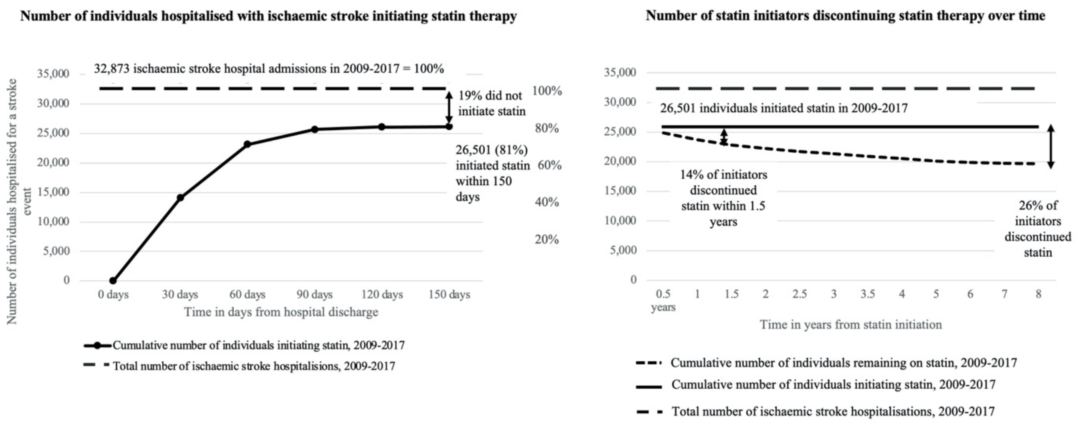

Thalmann I, *et al. Heart* 2023; 109:388–395. doi: 10.1136/heartjnl-2022-321452

Figure E Statin initiation and discontinuation rates over time following peripheral arterial disease event

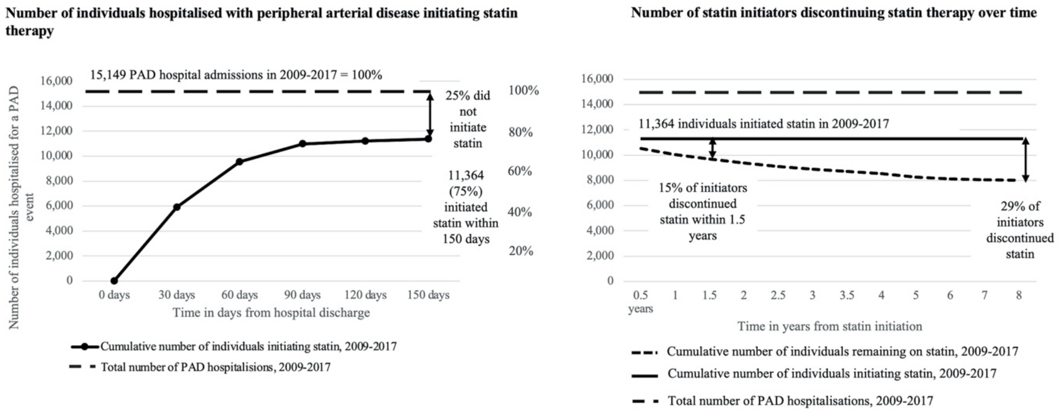

Figure F Statin initiation and discontinuation rates over time following other ASCVD events

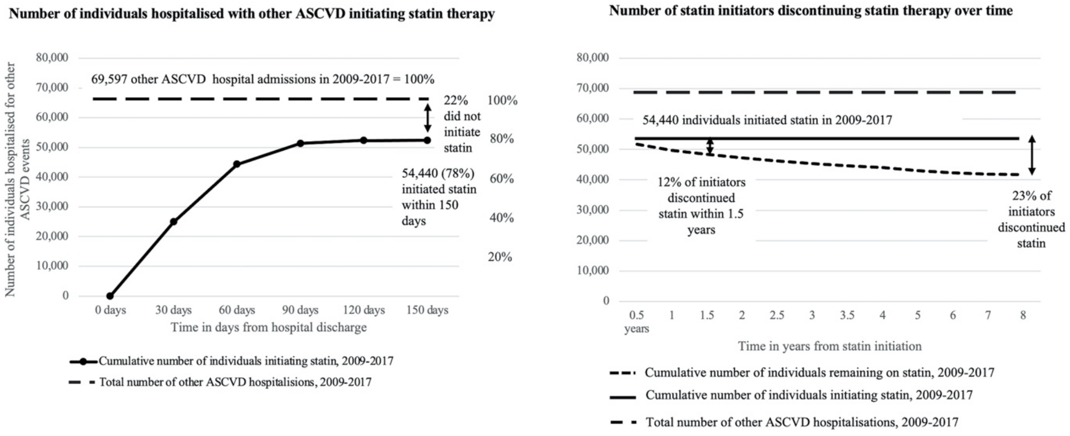

Thalmann I, *et al. Heart* 2023; 109:388–395. doi: 10.1136/heartjnl-2022-321452

Figure G Associations of patient characteristics with high-intensity statin initiation (a multivariable logistic regression model)

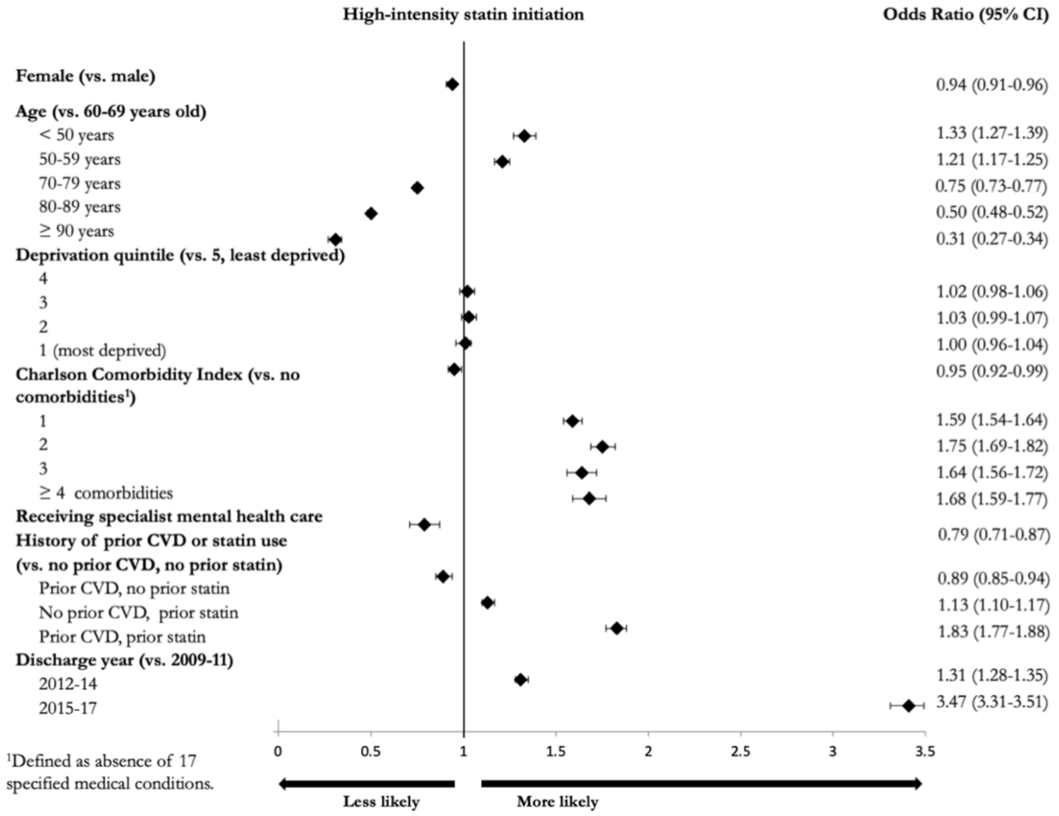

Please note that the Charlson Comorbidity Index (CCI) results presented in this figure are not easily interpretable due to index MI, stroke and PAD included among the CCI-eligible comorbidities, leaving the remaining index ASCVD conditions in the comparator "no comorbidity" category.
